# The roles of ethylene and transcription factors in the regulation of onset of leaf senescence

**DOI:** 10.3389/fpls.2014.00650

**Published:** 2014-11-25

**Authors:** Tomotsugu Koyama

**Affiliations:** Bioorganic Research Institute – Suntory Foundation for Life SciencesOsaka, Japan

**Keywords:** AP2/ERF, ethylene, leaf development, leaf senescence, NAC, transcription factor, TCP, WRKY

## Abstract

Leaf senescence is the last stage of leaf development and is accompanied by cell death. In contrast to senescence in individual organisms that leads to death, leaf senescence is associated with dynamic processes that include the translocation of nutrients from old leaves to newly developing or storage tissues within the same plant. The onset of leaf senescence is largely regulated by age and internal and external stimuli, which include the plant hormone ethylene. Earlier studies have documented the important role of ethylene in the regulation of leaf senescence. The production of ethylene coincides with the onset of leaf senescence, whereas the application of ethylene to plants induces precocious leaf senescence. Recently, many studies have described the components of ethylene signaling and biosynthetic pathways that are involved in modulating the onset of leaf senescence. Particularly, transcription factors (TFs) integrate ethylene signals with those from environmental and developmental factors to accelerate or delay leaf senescence. This review aims to discuss the regulatory cascade involving ethylene and TFs in the regulation of onset of leaf senescence.

## INTRODUCTION

**FIGURE 1:**
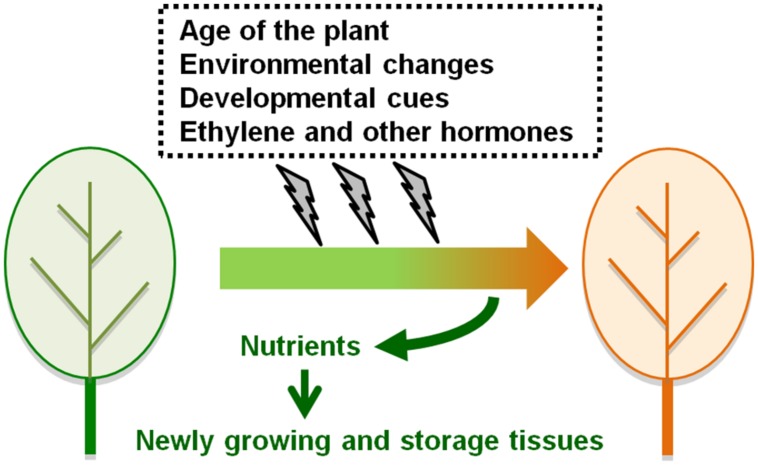
**The regulation of the onset of leaf senescence.** Leaf senescence occurs alongside color changes from green to yellow and brown. The onset of leaf senescence is affected by the age of the plant, but is also influenced by environmental conditions, developmental cues, ethylene, and other plant hormones. Nutrients derived from senescing leaves are translocated to newly growing or storage tissues within the same plant.

Leaf senescence occurs alongside color changes in leaves and is an easily visible phenomenon in the life cycle of a plant. Leaf senescence involves degradation of chlorophylls, carbohydrates, lipids, proteins, and nucleic acids and contributes to the mobilization of such nutrients from old leaves to growing or storage tissues. The importance of the efficient regulation of leaf senescence was reported by a study on the domestication of cultivated wheat. Loci tightly linked to the enrichment of several important nutrients in cereal grains encode transcription factors (TFs) that regulate the onset of leaf senescence in ancestral wheat plants (Uauy et al., 2006; Waters et al., 2009). The onset of leaf senescence is largely affected by the age of the plant, but is also influenced by changes in environmental conditions. Ethylene and other plant hormones accelerate or delay leaf senescence so that plants are better able to cope with severe environmental changes and achieve the maximum yield of seed and biomass production (Buchanan-Wollaston et al., 2003; Lim et al., 2007; **Figure 1**).

Upon leaf senescence, physiological events progress, which include chlorophyll breakdown, photosynthesis cessation, protein and nucleic acids degradation, catabolites and nutrients transport, and cell death responses, and the genes responsible for each event are dynamically up- or downregulated at the transcriptional level. Earlier studies have identified a group of senescence-associated genes (SAGs) that are induced upon senescence, and recent studies have shown specific roles for SAGs in leaf senescence (Gan and Amasino, 1997; Buchanan-Wollaston et al., 2005; Veyres et al., 2008). Indeed, treating plants with ethylene induces the expression of *SAG* genes (Jing et al., 2002). Dynamic changes in the expression profile of genes during leaf senescence can be visualized at the transcript and metabolite levels (Lin and Wu, 2004; Buchanan-Wollaston et al., 2005; van der Graaff et al., 2006; Balazadeh et al., 2008; Breeze et al., 2011; Watanabe et al., 2013).

Extensive transcriptome analysis revealed differential expression patterns of various families of TFs during leaf senescence (Lin and Wu, 2004; Buchanan-Wollaston et al., 2005; Breeze et al., 2011). Analysis of the promoters of differentially expressed genes during leaf senescence has found enrichment of certain TF motifs such as, NO APICAL MERISTEM, *Arabidopsis* TRANSCRIPTION ACTIVATION FACTOR, CUP-SHAPED COTYLEDON (NAC), APETALA2/ETHYLENE RESPONSE FACTOR (AP2/ERF), and WRKY families (Breeze et al., 2011). Genetic and molecular studies also provide strong evidence that the activities of NAC, AP2/ERF, WRKY, and several other TF family members influence the onset of leaf senescence (Buchanan-Wollaston et al., 2003; Lim et al., 2007). Significantly, ethylene modulates the activity of these TFs. These findings illustrate that ethylene-mediated modulation of TF activities underlie the onset of leaf senescence.

This review aims to provide a detailed overview of the regulatory cascade involving ethylene and TFs in the regulation of the onset of leaf senescence. This review first provides a brief overview of the role of ethylene in this process and then focuses on the detailed actions of NAC, AP2/ERF, WRKY, and other developmental regulators (**Table 1**). Emphasis is also placed on how ethylene modulates TF activities and interacts with other hormones during the development of leaf senescence.

**Table 1:** Transcription factors (TFs) regulating the onset of leaf senescence.

Name^a^	Accession number^b,c,d,e,f^	Family	Function^g^	Reference
ARF2^**a**^	AT5G62000^b^	ARF	Positive	Ellis et al. (2005), Lim et al. (2010)
NtERF3	D38124^d^	AP2/ERF	Positive	Koyama et al. (2013)
AtERF4	AT3G15210^b^	AP2/ERF	Positive	Koyama et al. (2013)
AtERF8	AT1G53170^b^	AP2/ERF	Positive	Koyama et al. (2013)
SlERF36	SGN-U564952^c^	AP2/ERF	Positive	Upadhyay et al. (2013)
RAV1	AT1G13260^b^	AP2/ERF	Positive	Woo et al. (2010)
GmRAV	NM_001250671^d^	AP2/ERF	Positive	Zhao et al. (2008)
EDF1	AT1G25560^b^	AP2/ERF	Negative	Chen et al. (2011)
EDF2	AT1G68840^b^	AP2/ERF	Negative	Chen et al. (2011)
SUB1A	LOC_Os09g11480^e^	AP2/ERF	Negative	Fukao et al. (2012)
CBF2	AT4G25470^b^	AP2/ERF	Negative	Sharabi-Schwager et al. (2010)
CBF3	AT4G25480^b^	AP2/ERF	Negative	Sharabi-Schwager et al. (2010)
CRF6	AT3G61630^b^	AP2/ERF	Negative	Zwack et al. (2013)
CIB	Glyma11g12450^f^	bHLH	Positive	Meng et al. (2013)
EIN3	AT3G20770^b^	EIN3	Positive	Li et al. (2013), Kim et al. (2014)
GLK2	AT5G44190^b^	GARP	Negative	Rauf et al. (2013)
GBF1	AT4G36730^b^	GBF	Positive	Smykowski et al. (2010)
GAI^a^	AT1G14920^b^	GRAS	Negative	Chen et al. (2014)
GRF3	AT2G36400^b^	GRF	Negative	Debernardi et al. (2014)
Knotted1	AY312169^d^	homeodomain	Negative	Ori et al. (1999)
KNAT2	AT1G70510^b^	homeodomain	Negative	Hamant et al. (2002)
FYF	AT5G62165^b^	MADS	Negative	Chen et al. (2011)
MYBR1/MYB44	AT5G67300^b^	MYB	Negative	Jaradat et al. (2013)
NAM-B1	DQ871219^d^	NAC	Positive	Uauy et al. (2006)
AtNAP	AT1G69490^b^	NAC	Positive	Guo and Gan (2006), Zhang and Gan (2012)
ORE1	AT5G39610^b^	NAC	Positive	Kim et al. (2009)
ANAC019	AT1G52890^b^	NAC	Positive?	Hickman et al. (2013)
ANAC055	AT3G15500^b^	NAC	Positive?	Hickman et al. (2013)
OsNAP	LOC_Os03g21060^e^	NAC	Positive	Zhou et al. (2013), Liang et al. (2014)
ORS1	AT3G29035^b^	NAC	Positive	Balazadeh et al. (2011)
VNI2	AT5G13180^b^	NAC	Negative	Yang et al. (2011)
JUB1^a^	AT2G43000^b^	NAC	Negative	Wu et al. (2012)
TCP2	AT4G18390^b^	TCP	Positive	Schommer et al., 2008
TCP3	AT1G53230^b^	TCP	Positive	Schommer et al. (2008), Koyama et al. (2013)
TCP4	AT3G15030^b^	TCP	Positive	Schommer et al. (2008), Koyama et al. (2013)
TCP5	AT5G60970^b^	TCP	Positive	Koyama et al. (2013)
TCP10	AT2G31070^b^	TCP	Positive	Schommer et al. (2008), Koyama et al. (2013)
TCP13	AT3G02150^b^	TCP	Positive	Koyama et al. (2013)
TCP19	AT5G51910^b^	TCP	Negative	Danisman et al. (2012)
TCP20	AT3G27010^b^	TCP	Negative	Danisman et al. (2012)
TCP24	AT1G30210^b^	TCP	Positive	Schommer et al. (2008)
WRKY6	AT1G62300^b^	WRKY	Positive	Robatzek and Somssich (2002)
WRKY53	AT4G23810^b^	WRKY	Positive	Miao and Zentgraf (2007)
WRKY54	AT2G40750^b^	WRKY	Negative	Besseau et al. (2012)
WRKY57	AT1G69310^b^	WRKY	Negative	Jiang et al. (2014)
WRKY70	AT3G56400^b^	WRKY	Negative	Besseau et al. (2012)
SlZF2	ADZ15317^d^	Zn finger	Negative	Hichri et al. (2014)

*^a^Abbreviations: GIBBERELLIC ACID INSENSITIVE (GAI) and JUNGBRUNNEN1 (JUB1). Other TF names are defined in the main text. ^b,c,d,e,f^Accession numbers: The sequence data can be found in ^b^*Arabidopsis* Genome Initiative, ^c^Sol genomic network, ^d^Genbank, ^e^Michigan State University Rice Genome Annotation Project, and ^f^Phytozome libraries. ^g^Function: Positive and negative indicate TFs that accelerate and delay leaf senescence, respectively.*

## ETHYLENE AS A REGULATOR OF THE ONSET OF LEAF SENESCENCE

Earlier studies reported the involvement of ethylene in the regulation of leaf senescence. Ethylene production is associated with the onset and progression of leaf senescence in various plant species (Abel et al., 1992). Application of ethylene to leaves stimulates senescence, but inhibitors of ethylene perception or biosynthesis delay leaf senescence (Aharoni and Lieberman, 1979; Kao and Yang, 1983). Furthermore, downregulation of an ethylene biosynthesis gene in tomato plants led to a decrease in ethylene production and substantially delayed leaf senescence, clearly suggesting that ethylene, produced as plants age, accelerates leaf senescence (John et al., 1995).

**FIGURE 2:**
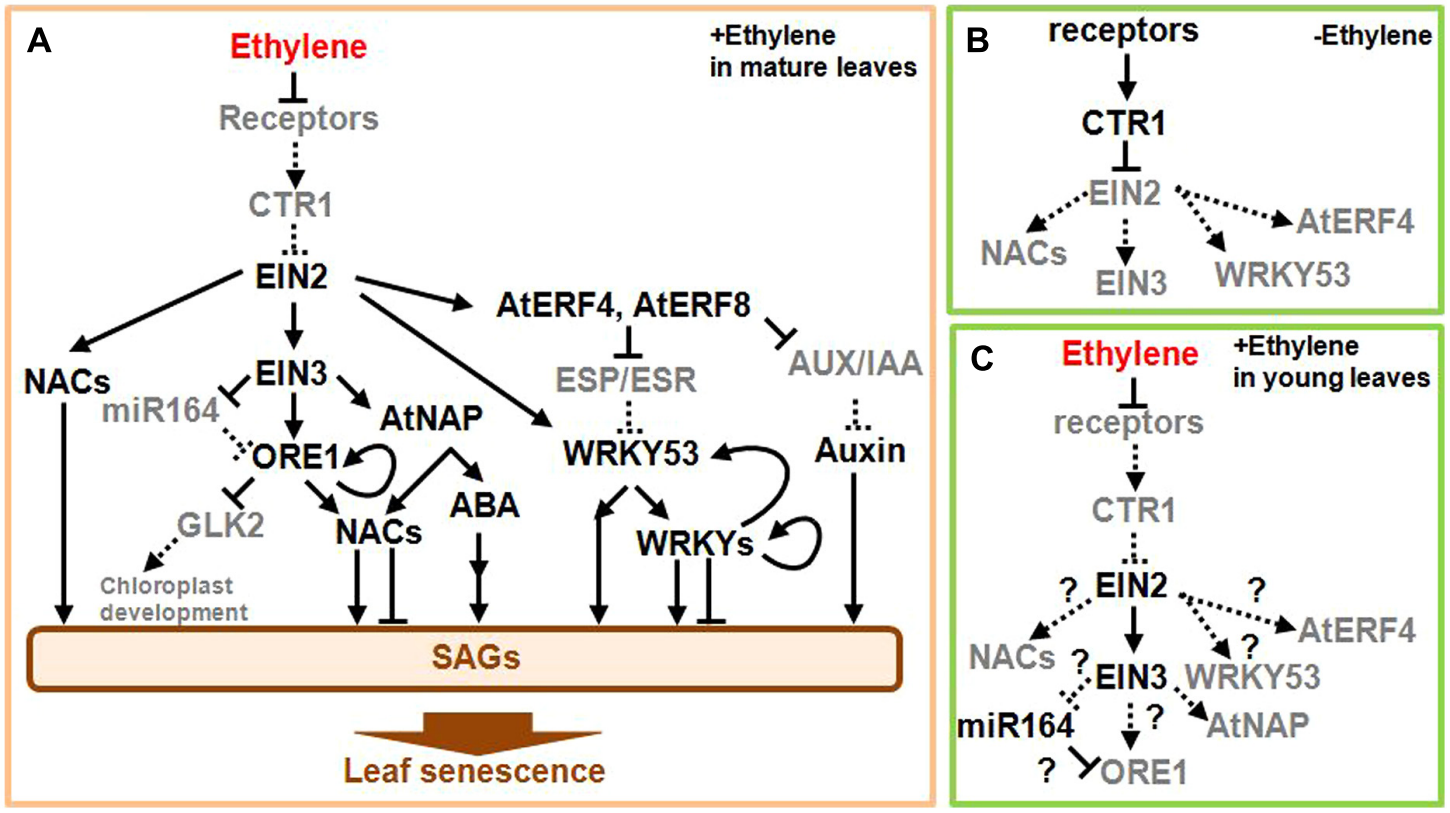
**Scheme of the ethylene signaling pathway leading to the onset of leaf senescence. (A)** In mature leaves, the detection of ethylene activates the downstream signaling pathway leading to SAG induction and leaf senescence. **(B)** In young and mature leaves, the receptors constitutively repress the downstream signaling in the absence of ethylene. **(C)** In young leaves, the detection of ethylene activates the downstream signaling pathway, but does not itself induce leaf senescence. Note that EIN2 and EIN3 are active and induce some ethylene responses, but not leaf senescence by an uncharacterized mechanism, in which some regulators of leaf development are likely involved. Arrows and bars at the end of each line show positive and negative regulations, respectively. Solid lines and black gene names designate the active form, while dotted lines and gray gene names indicate the inactive form. Several transcription factors (TFs) and signals such as jasmonic acid are not drawn in this scheme owing to space limitations. A detailed description on the scheme is presented in the main text.

Knowledge of the ethylene signaling pathway will help to clarify the regulatory gene network involved in the onset of leaf senescence. As shown in **Figure 2A**, receptors localized on the endoplasmic reticulum (ER) membrane detect ethylene (Kendrick and Chang, 2008). Since these receptors repress the activity of downstream signaling components in the absence of ethylene (**Figure 2B**), ethylene reverses this repression and thus activates the signaling pathway. The signal generated following the detection of ethylene is subsequently transmitted to a complex composed of CONSTITUTIVE TRIPLE RESPONSE1 (CTR1), a Raf-like serine/threonine protien kinase, and ETHYLENE INSENSITIVE2 (EIN2), which is an integral ER membrane protein (Ju et al., 2012; Qiao et al., 2012). In the absence of the ethylene signal, CTR1 directly phosphorylates the cytosolic carboxyl-terminal domain of EIN2 (EIN2-C), whereas the ethylene signal prevents this phosphorylation and results in cleavage of EIN2-C, which then translocates to the nucleus and activates ETHYLENE-INSENSITIVE3 (EIN3) and EIN3-LIKE (EIL) TFs. The ethylene signal stabilizes EIN3 and EIL TFs, which are short-lived proteins in the absence of ethylene (Guo and Ecker, 2003; Potuschak et al., 2003), consequently inducing various physiological responses including the onset of leaf senescence.

Mutations in components of the ethylene signaling pathway exhibit differential timing of the onset of senescence, clearly suggesting that these components are involved in the regulation of such process. Consistent with the repressive role of ethylene receptors including ETHYLENE RESISTANT1 (ETR1) in the signaling pathway, a dominant-negative version of the receptors, such as the *etr1* mutation, delays leaf senescence in *Arabidopsis* and petunia plants (Grbić and Bleecker, 1995; Wang et al., 2013). In contrast, an *Arabidopsis* null mutant that lacks two of five ethylene receptor genes has a phenotype consistent with constitutive ethylene response as well as accelerated leaf senescence (Qu et al., 2007). A pivotal role of EIN2 in the positive regulation of leaf senescence was documented by characterizing the genetic loci controlling the onset of leaf senescence in *Arabidopsis* (Oh et al., 1997; Kim et al., 2009). EIN3 positively regulates the onset of leaf senescence, since the *ein3* mutant delays leaf senescence whereas overexpression of *EIN3* gene accelerates it (Li et al., 2013; Kim et al., 2014). In contrast, the *ctr1* mutant does not induce precocious leaf senescence and the involvement of CTR1 in the regulation of leaf senescence remains unclear. (Jing et al., 2005).

## ETHYLENE-REGULATED NAC AND OTHER TFs CONTROL THE ONSET OF LEAF SENESCENCE

Several reports have attempted to elucidate the mechanism through which the ethylene signaling pathway modulates NAC activities during the onset of leaf senescence (Kim et al., 2009, 2014; Li et al., 2013; **Figure 2A**). The NAC TF family includes 105 members in *Arabidopsis* that are important during development and stress responses (Mitsuda and Ohme-Takagi, 2009). Among *NAC* genes upregulated during leaf senescence, six *NAC* genes including *ORESARA1 (ORE1)/ANAC092, ANAC019, NAC-like activated by AP3 (AtNAP), ANAC047, ANAC055,* and *ORE1 SISTER1 (ORS1)/ANAC059* are activated through the *EIN2*-dependent pathway (Kim et al., 2009, 2014; **Figure 2A**). ORE1 positively regulates the onset of leaf senescence and activates the expression of *ORE1* itself, other *NAC*, nuclease, a sugar transporter, and various *SAG* genes (Kim et al., 2009; Balazadeh et al., 2010; Breeze et al., 2011; Matallana-Ramirez et al., 2013; Rauf et al., 2013). ORE1 interacts with GOLDEN-LIKE2 (GLK2), the GARP family TF required for chloroplast development (Rauf et al., 2013). ORE1 attenuates GLK2 activity and may stop the maintenance of chloroplast development. ORE1 activity is modulated at both transcriptional and post-transcriptional levels (Kim et al., 2009; **Figure 2A**). *ORE1* mRNA is targeted by the micro RNA *miR164*. The decrease in *miR164* content with leaf aging is largely dependent on the *EIN2* gene and thus leads to the accumulation of *ORE1* mRNA in old leaves. Recent studies have further revealed that EIN3 directly activates expression of *ORE1* (Li et al., 2013; Kim et al., 2014). Interestingly, EIN3 represses three *miR164* precursor genes and is also involved in both positive and indirect regulation of the *ORE1* gene (Li et al., 2013). Consistent with the molecular evidence, *ORE1* expression is reduced in *ein3* mutant during leaf senescence. These observations suggest that EIN3, miR164, and ORE1 comprise a regulatory network that operates downstream of the ethylene signaling pathway (**Figure 2A**).

Among other *NAC* genes downstream of EIN2, the *AtNAP* gene is under the direct control of EIN3, whereas *ORS1, ANAC019, ANAC047*, and *ANAC055* genes are activated in an EIN3-independent manner (Kim et al., 2014; **Figure 2A**). AtNAP positively regulates the onset of leaf senescence and activates a component of the abscisic acid (ABA) signaling pathway, which promotes both leaf senescence and stress responses (Guo and Gan, 2006; Zhang and Gan, 2012). A rice homolog gene, *OsNAP1*, acts as a positive regulator of leaf senescence and its product directly targets an ABA biosynthesis enzyme gene (Zhou et al., 2013; Liang et al., 2014). ORS1 positively regulates the onset of leaf senescence (Balazadeh et al., 2011). ANAC019 and ANAC055 seem to function under the control of C-REPEAT/DEHYDRATION RESPONSIVE ELEMENT BINDING FACTORS (CBFs) of AP2/ERF TFs and other TFs during stress response and leaf senescence (Hickman et al., 2013; See below). A role for ANAC047 in leaf senescence is yet to be determined.

Five additional *NAC* genes including *VND-INTERACTING2 (VNI2)* are thought to function downstream of ORE1 and AtNAP (Kim et al., 2014). VNI2 negatively regulates the onset of leaf senescence via the direct activation of *COLD REGULATED (COR)* and *RESPONSIVE TO DEHYDRATION (RD)* genes that are also responsive to environmental stimuli (Yang et al., 2011). By contrast, the functions of other NAC genes remain to be clarified. The regulation of EIN2, EIN3, NAC TFs, and the ABA response pathway are likely to be important in the integration of various inputs from diverse environmental factors as well as the age of the plant (**Figure 2A**).

## ETHYLENE-RESPONSIVE TFs IN THE REGULATION OF ONSET OF LEAF SENESCENCE

Ethylene activates a substantial number of *AP2/ERF* genes, and several of these regulate the onset of leaf senescence. The AP2/ERF TFs comprise 146 members that include both activators and repressors of transcription (Mitsuda and Ohme-Takagi, 2009). A subgroup of transcriptional repressors with the ERF-associated repression (EAR) motif, such as NtERF3, AtERF4, and AtERF8, positively regulate the onset of leaf senescence in *Arabidopsis* (Ohta et al., 2001; Koyama et al., 2013; **Figure 2A**). The finding of *EIN2*-dependent *AtERF4* expression in leaves suggests that there is AtERF4 activity downstream of EIN2 (Fujimoto et al., 2000). AtERF4 and AtERF8 are degraded by a proteasomal-dependent pathway, but accumulate within the plant as a result of increasing age (Koyama et al., 2013). These ERF TFs directly repress expression of the *EPITHIOSPECIFIER PROTEIN/EPITHIOSPECIFYING SENESCENCE REGULATOR (ESP/ESR)* gene, a negative regulator of the onset of leaf senescence (Miao and Zentgraf, 2007; Koyama et al., 2013). The *ESP/ESR* transcript is highly expressed in young leaves, but decreased in old ones (Koyama et al., 2013). ESP/ESR inhibits the activity of WRKY53, a positive regulator of the onset of leaf senescence, at both transcriptional and post-translational levels (Miao and Zentgraf, 2007; See below). These findings imply that AtERF4 and AtERF8 activate WRKY53 by removing the *ESP/ESR*-mediated inhibition. Therefore, AtERF4, AtERF8, ESP/ESR, and WRKY53 form another regulatory network for the onset of leaf senescence (**Figure 2A**). Moreover, AtERF4 and AtERF8 repress the expression of *AUXIN/INDOLE-3-ACETIC ACID (AUX/IAA)* genes. AUX/IAA TFs generally suppress auxin responses that include positive effects on leaf senescence. Therefore, it is possible that the AtERF4- and AtERF8-mediated *AUX/IAA* repression enhances auxin response and then stimulates the onset of leaf senescence. In addition, a tomato homolog of *AtERF4*, *SlERF36,* accelerates leaf senescence when overexpressed in tomato plants (Upadhyay et al., 2013).

By contrast, RAV1 and GmRAV1, which possess another type of repression domain (Ikeda and Ohme-Takagi, 2009), negatively regulate the onset of leaf senescence, because overexpression of these *RAV1* genes delays leaf senescence in *Arabidopsis* (Woo et al., 2010). Other two *Arabidopsis RAV* genes, namely, *ETHYLENE RESPONSE DNA BINDING FACTOR1 (EDF1)* and *EDF2*, are proposed to regulate the onset of leaf senescence downstream of the MADS box TF, FOREVER YOUNG FLOWER (FYF; Chen et al., 2011). These *RAV* genes are transcriptionally induced by ethylene (Alonso et al., 2003). Based on studies investigating EAR- and RAV-type AP2/ERF TFs, the ethylene signal appears to balance positive and negative regulations thus determining the rate of leaf senescence.

Transcriptional activators of ERF TFs are also involved in regulating the onset of leaf senescence. SUBMERGENCE1A (SUB1A) negatively regulates the onset of leaf senescence in rice (Fukao et al., 2012), while CYTOKININ RESPONSE FACTOR6 (CRF6) negatively regulates leaf senescence (Zwack et al., 2013). Overexpression of *CBF2* and *CBF3* genes delays leaf senescence in *Arabidopsis* (Sharabi-Schwager et al., 2010). Since CBFs target *COR15* and *RD29* genes and possibly control ANAC019 and ANAC055 (Yang et al., 2011; Hickman et al., 2013), CBFs seem to regulate the onset of leaf senescence via these downstream genes. However, there have been no reports on the involvement of ethylene in the regulation of these ERF activators.

## WRKY TFs INTEGRATE ETHYLENE AND JASMONIC ACID SIGNALS DURING LEAF SENESCENCE

Jasmonic acid (JA) is another important factor regulating the onset of leaf senescence, because a mutant that lacks a JA-biosynthetic enzyme gene delays leaf senescence and application of JA to leaves accelerates senescence (He et al., 2002; Seltmann et al., 2010). JA often cooperatively interacts with ethylene to underpin many physiological responses (Lorenzo et al., 2004; Zhu et al., 2011). It has been well documented that JA, along with the age of the plant, induce transcription of many *WRKY* genes. (Lin and Wu, 2004). Among WRKY TFs activated by JA, WRKY53 positively regulates the onset of leaf senescence and its activity is modulated by ESP/ESR at both transcriptional and post-translational levels (Miao and Zentgraf, 2007; **Figure 2A**). ESP/ESR physically interacts with WRKY53 and, presumably, prevents WRKY53 binding to DNA. ESP/ESR also inhibits the accumulation of *WRKY53* transcripts in leaves. It has also been reported that WRKY6, WRKY54, WRKY57, and WRKY75 regulate leaf senescence (Robatzek and Somssich, 2002; Besseau et al., 2012; Li et al., 2012b). Since WRKY6 and WRKY53 have been shown to increase many *WRKY* transcripts in addition to *SAGs* (Robatzek and Somssich, 2002; Miao et al., 2004), some self-amplification of WRKY activity may contribute to the robust control of leaf senescence.

Several reports provide intriguing insights into the interactions between WRKY TFs and the ethylene signaling pathway. Nematode-induced WRKY53 expression in leaves requires a functional *EIN2* gene, suggesting the involvement of EIN2 in the modulation of WRKY53 activity (Murray et al., 2007). WRKY75 is also involved in the ethylene-dependent defense-signaling pathway (Chen et al., 2013). WRKY33, whose transcript is markedly increased during leaf senescence, directly activates ethylene biosynthetic genes and is involved in ethylene production (Breeze et al., 2011; Li et al., 2012a). Thus, it is possible that WRKY TFs are activated by the cooperative action of the ethylene and JA signaling pathways in the regulation of the onset of leaf senescence.

In addition, the single stranded-DNA binding protein WHIRLY1A and a histone methyltransferase target the *WRKY53* gene in *Arabidopsis* (Ay et al., 2009; Miao et al., 2013). A basic loop-helix-loop TF activates the *WRKY53* gene and positively regulates the onset of leaf senescence in soybean (Meng et al., 2013). However, an association between ethylene and these WRKY53 regulators remains to be addressed.

## THE ONSET OF LEAF SENESCENCE IS AFFECTED BY TFs THAT ARE REQUIRED FOR LEAF DEVELOPMENT

In contrast to the pivotal role of ethylene in the onset of leaf senescence, it is known that ethylene inhibits leaf expansion in young plants, but does not always induce leaf senescence (Kieber et al., 1993; Hua and Meyerowitz, 1998). Moreover, *Arabidopsis ctr1* mutant has constitutive responses to ethylene resulting in the formation of a small rosette, but does not appear to induce precocious leaf senescence (Kieber et al., 1993; Jing et al., 2005). These apparent discrepancies suggest that the mechanism leading to leaf senescence might require some developmental regulators even when the ethylene signaling pathway is activated (**Figure 2C**). Whereas the functional interaction between ethylene and developmental regulators during leaf senescence is not fully understood, it is expected that such regulators have pivotal roles in the onset of leaf senescence.

KNOTTED1-like homeodomain (KNOX) TFs, which are required for shoot meristem and leaf development, negatively regulate the onset of leaf senescence (Ori et al., 1999; Hamant et al., 2002; Hay and Tsiantis, 2010). When *KNOX* genes are ectopically expressed in tobacco and *Arabidopsis* leaves, they markedly delay the onset of leaf senescence (Ori et al., 1999; Hamant et al., 2002). Ectopic *KNOX* expression confers an undifferentiated cell fate in leaves and inhibits their differentiation (Hay and Tsiantis, 2010). As a consequence, this disordered cellular regulation may indirectly delay the onset of leaf senescence. Otherwise, KNOX TFs regulate biosynthetic genes of cytokinin (Sakamoto et al., 2001; Hay et al., 2002), which acts as a negative regulator of the onset of leaf senescence, and therefore, possibly influences leaf senescence. In agreement with the antagonism between *KNOX* genes and the gibberellin (GA) signaling pathway observed in shoot meristem and leaf development (Hay and Tsiantis, 2010), plants treated with GA and *Arabidopsis* mutants of the GRAS-type TF genes, which are negative regulators of the GA signaling pathway, accelerate leaf senescence (Chen et al., 2014).

Another class of regulators for both leaf senescence and development is the TEOSINTE BRANCHED1, CYCLOIDEA, PCNA BINDING FACTOR (TCP) TFs family. A combined analysis of high-resolution temporal clustering of genes differentially expressed during leaf senescence and TF-binding motif searching in the promoters of each cluster demonstrates that the TCP-binding motif is significantly enriched in certain downregulated gene clusters (Breeze et al., 2011). This indicates the co-regulation of these gene clusters and TCP activity. Consistent with bioinformatic surveillance, reverse genetic analysis revealed that inhibition of the *CINCINNATA (CIN)* subfamily of *TCP* (*CIN*-like *TCP*) delays leaf senescence whereas overexpression of a *CIN*-like *TCP* gene accelerates it (Schommer et al., 2008; Koyama et al., 2013). A possible scenario to explain the positive roles of CIN-like TCP TFs in the onset of leaf senescence is that CIN-TCP TFs activate JA biosynthetic enzyme genes (Schommer et al., 2008). Alternatively, CIN-like TCP TFs suppress an auxin signaling pathway, which is a negative regulator of leaf senescence, and also activates negative regulators of *KNOX* genes (Koyama et al., 2007, 2010). Moreover, CIN-like TCPs act as heterochronic regulators of leaf development and consequently influence the onset of leaf senescence (Efroni et al., 2008). By contrast, TCP19 and TCP20, which are grouped into a class I subgroup, negatively regulate the onset of leaf senescence and results in the opposite effects of CIN-like TCPs (Danisman et al., 2012).

In addition to KNOX and TCP TFs, *Arabidopsis* GROWTH-REGULATING FACTOR (GRF) TFs and a tomato C2H2 type-EAR repressor regulate both leaf development and senescence (Debernardi et al., 2014; Hichri et al., 2014). Taking the roles of the developmental regulators into account, these regulators, thus, prevent precocious leaf senescence. Ethylene meditates various signals required for the induction of defense responses against biotic and abiotic stressors (Kendrick and Chang, 2008); however, these responses are not always followed by cell death. Therefore, such developmental regulators are likely to determine the fate of leaves upon ethylene exposure. In comparison to fully maturated leaves, young leaves accumulate low amounts of carbon and nitrogen sources that would be mobilized to growing and storage organs and therefore it is reasonable that young leaves are kept away from senescence even in the presence of ethylene.

## CONCLUSIONS AND PERSPECTIVES

In addition to ethylene, JA and the developmental signals discussed in this review, additional factors such as cytokinin, auxin, ABA, and hydrogen peroxide are involved in the regulation of leaf senescence (Ellis et al., 2005; Lim et al., 2010; Smykowski et al., 2010; Yang et al., 2011; Wu et al., 2012; Jaradat et al., 2013). Several TFs are reported to regulate the onset of leaf senescence under these additional signals and details of such TF are listed in **Table 1**. Ethylene and these signals are integrated for the regulation of the onset of leaf senescence; however, there have been no reports of a direct interaction between ethylene and such TFs acting downstream of these signals. It is interesting to investigate whether these TFs act in an ethylene-dependent manner during the onset of leaf senescence.

This review focuses on the roles of TFs and ethylene in the regulation of the onset of leaf senescence and emphasizes that regulation occurs at multiple levels downstream of the ethylene signaling pathway. Moreover, leaf development is tightly linked to the onset of senescence and further clarification of such mechanisms is in progress. Furthermore, the effect of ethylene on the stimulation of leaf senescence is dependent on the duration of ethylene exposure (Jing et al., 2005). Regulation of the appropriate duration of ethylene exposure could represent another candidate for modulating the ethylene signal and thus, the onset of leaf senescence. Further efforts to determine the mechanism that transforms the ethylene signal into the onset of leaf senescence will improve our current understanding of the roles of ethylene in leaf senescence.

## Conflict of Interest Statement

The author declares that the study was conducted in the absence of any financial, commercial or other relationships that might be perceived by the academic community as representing a potential conflict of interest.
